# The Drug Habits and Their Treatment

**Published:** 1902-07

**Authors:** 


THE DRUG HABITS AND THEIR TREATMENT. A Clinical Summary of Some of the General Facts Recorded in Practice. By T. D. CROTHERS, M. D., Superintendent Walnut Lodge Hospital, Hartford, Conn.; Professor Diseases of the Brain and Nervous System, New York School of Clinical Medicine. Pp. 96. Chicago: G. P. Englehard & Co. 1902. [Price, $1.00 net.]

For many years the author of this little work has been doing pioneer work in the field of dipsomania and no one has disputed his title to "the leading authority" in this country on the disease of inebriety. A work from his pen is worthy of careful reading and consideration and must carry conviction even to unbelievers. The book is divided into four chapters: I. Habit and damages of alcohol; II. Dipsomania; III. Opium inebriety; IV. Cocain and other drug habits. The increasing demand for stimulants of this order makes it imperative for physicians to be alive to the dangers of their overuse, and to know how to direct treatment when these cases fall into their hands. This is the object of the book. W.C.K.

